# Left Atrial Appendage Thrombus Formation in a Patient on Dabigatran Therapy Associated With *ABCB1* and *CES-1* Genetic Defect

**DOI:** 10.3389/fphar.2018.00491

**Published:** 2018-05-15

**Authors:** Zhi-Chun Gu, Xiao-Wei Ma, Xiao-Yuan Zheng, Long Shen, Fang-Hong Shi, Hao Li

**Affiliations:** ^1^Department of Pharmacy, Renji Hospital, School of Medicine, Shanghai Jiaotong University, Shanghai, China; ^2^Department of Clinical Laboratory, Renji Hospital, School of Medicine, Shanghai Jiaotong University, Shanghai, China; ^3^Department of Pharmacy, Affiliated Hospital of Jiangnan University, Wuxi, China; ^4^Department of Cardiology, Renji Hospital, School of Medicine, Shanghai Jiaotong University, Shanghai, China; ^5^Department of Pharmacy, Shanghai Children's Medical Center, School of Medicine, Shanghai Jiaotong University, Shanghai, China

**Keywords:** dabigatran, left atrial appendage thrombus, genetic polymorphism, ABCB1, CES-1, drug-drug interaction

## Abstract

Dabigatran, directly targeting thrombin, is widely used for the prevention of stroke in nonvalvular atrial fibrillation (NVAF). We reported a rare case of left atrial appendage thrombus formation in a persistent NVAF patient despite the 31 months uninterrupted treatment with dabigatran 110 mg twice daily. The patient is a carrier of ABCB1 variant alleles with 7 heterozygote single nucleotide polymorphisms (SNPs: rs4148738, rs2235046, rs1128503, rs10276036, rs1202169, rs1202168, rs1202167) as well as CES-1 variant alleles with 2 homozygote SNPs (rs2244613 and rs4122238) and 2 heterozygote SNPs (rs8192935 and rs4580160), which may contribute to the changes of dabigatran plasma concentration. In addition, Drug-drug interaction with atorvastatin may also play a role to decrease dabigatran plasma concentration. There are only four such cases till date, of which had thrombus in the left atrium, reported in the literature. We firstly reported the documented case in a Chinese patient carrying multiple alleles of ABCB1 and CES-1, who suffered from thrombus in the left atrial appendage despite long-term anticoagulation with dabigatran. More clinical data are required to elucidate the impact of CES-1 and ABCB1 polymorphism on dabigatran pharmacokinetics, especially for Asian.

## Introduction

Warfarin, one of the vitamin K-dependent antagonists (VKAs), is the most commonly used oral anticoagulant (Mega and Simon, 2015). Although warfarin has been used clinically for more than 60 years, several challenges including bleeding complications have been noted, which are one of the primacy causes of severe adverse drug events (Crowther et al., 2000; Wysowski et al., 2007). The inherent limitations of warfarin, comprising narrow therapeutic window, intra-patient variability, and numerous food-drug interaction and drug-drug interaction, lead to a need for more elaborative anticoagulation monitoring (Johnson et al., 2011; Pirmohamed et al., 2013). Different from warfarin, non-VKA oral anticoagulants (NOACs), which directly target thrombin or Xa factor, have been approved for the prevention of stroke and systemic embolism in non-valvular atrial fibrillation without necessary of routine blood monitoring (López-López et al., 2017). Dabigatran etexilate, the prodrug of dabigatran, has the lowest bioavailability of present NOACs of 3~7% (Mega and Simon, 2015), which is rapidly converted by esterases-1 (*CES-1*) to dabigatran after administration (Stangier and Clemens, 2009; Laizure et al., 2014). In addation, dabigatran etexilate is a substrate of the P-glycoprotein intestinal efflux transporter (*P-gp*), which also known as ATP-binding cassette sub-family B member 1 (*ABCB1*) (Paré et al., 2013). P-gp is one of the drug transporters expressed in gastrointestinal tract which involves in the efflux of various kinds of drugs into lumen (Ambudkar et al., 2003). Thus, strong P-gp inhibitors can increase dabigatran bioavailability by 12–23% (Liesenfeld et al., 2011; Paré et al., 2013; Kishimoto et al., 2014). Unlike Xa factor inhibitors, oxidoreductases or cytochrome P450 enzymes are not involved in the metabolism of dabigatran (Blech et al., 2008). As the renal clearance of dabigatran is 80%, renal function should be monitored regularly in patients taking dabigatran (Mega and Simon, 2015). Accordingly, changes in the process of absorption or elimination could have a great effect on dabigatran plasma concentrations (Antonijevic et al., 2017; Favaloro et al., 2017). We reported a dabigatran-treated patient who presented with severe left atrial appendage thrombus formation, of whom *ABCB1* and *CES1* genetic polymorphism and drug-drug interaction may have been the contributing factors. The patient gave his written informed consent for publication of this report.

## Case presentation

**Figure 1:**
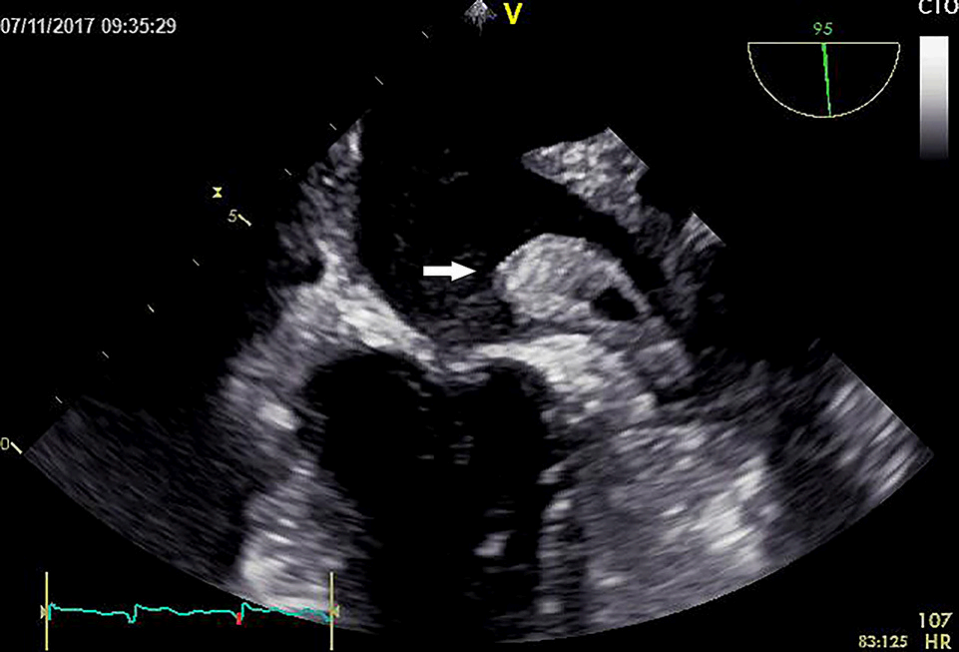
Transesophageal echocardiogram showed the large left atrial thrombus.

The present patient is a 70-year-old Chinese male with paroxysmal atrial fibrillation, complicating hypertension, type 2 diabetes mellitus, coronary heart disease, cerebral infarction, and lost binocular vision. The patient had received dabigatran etexilate 110 mg twice daily since April 30th, 2015 for the prevention of stroke and systemic embolism. Before the introduction of dabigatran, several drugs, including atorvastatin, have been taking for years (Table 1). The patient was admitted to the department of cardiology on November 9th 2017 due to the recurrence of cardio-embolic stroke. Vital signs were normal with blood pressure of 133/73 mmHg and heart rate of 73 rates per minute. Transesophageal echocardiography on admission showed a large thrombus in the left atrial appendage (Figure 1, Video 1), the enlarged left atrial diameter of 46 mm (reference: 30–40 mm) as well as depressed left ventricular function with left ventricular ejection fraction of 52% (reference: 55%) (Table 2). Laboratory data on admission were within normal limits expect for serum creatinine of 115 umol/L (reference: 45–104 umol/L) and B type natriuretic peptide (BNP) of 188 pg/mL (reference: 0.0~100 pg/mL) (Table 2). Dabigatran was stopped at admission, and enoxaparin was administrated with the combination of warfarin since November 8th, 2017 for 20 days till November 28th, 2017. Finally, the patient was treated with coronary artery bypass grafting (CABG), atrial fibrillation ablation, left atrial appendage excision and pericardial drainage plus cardiac temporary pacemaker implantation in November 29th, 2017. He was discharged day 15 post-operation on treatment with warfarin 2.5 mg daily. At 3 months follow up, the patient has been doing well without any evidence of recurrent thrombotic events.

**Table 1:** Drugs administration during 3 years.

**Starting Time**	**June 21st, 2014**	**Nov. 30th, 2014**	**Apr. 4th, 2015**	**Nov. 11th, 2017**
Stop Time	Nov. 29th, 2014	Apr. 3rd, 2015	Nov. 8th, 2017	Dec. 20th, 2017
Fosinopril Sodium Tablets	NK	–	–	–
Amlodipine Besylate Tablets	5 mg bid	5 mg bid	5 mg bid	–
Metoprolol Tartrate Tablets	NK	25 mg bid	25 mg bid	–
Clopidogrel Hydrogen Sulphate Tablets	–	75 mg qd	–	–
**Atorvastatin Tablets**	–	20 mg qd	**20 mg qd**	–
Metformin Hydrochloride Tablets	–	500 mg qd	500 mg qd	–
**Dabigatran Etexilate Capsules**	–	–	**110 mg bid**	–
Clonidine Hydrochloride Tablets	–	–	75 μg qd	–
Olmesartan Medoxomil	–	–	20 mg qd	–
Warfarin Tablets	–	–	–	2.5 mg qn

*NK, not known*.

**Table 2:** Results of main laboratory test.

**Index (normal range)**	**Nov. 8th**	**10th**	**12th**	**14th**	**16th**	**20th**	**23th**	**27th**	**29th**	**30th**
**REGULAR BLOOD ANALYSIS**										
White blood cell count (3.97~9.15 × 10^∧^9/L)	8.53	–	–	–	–	–	–	–	15.65	24.86
Neutrophils% (50~70%)	54.8	–	–	–	–	–	–	–	85.0	89.9
Platelet count (85~303 × 10^∧^9/L)	200	–	–	–	–	–	–	–	133	121
Red blood cell count (4.09~5.74 × 10^∧^12/L)	5.56	–	–	–	–	–	–	–	3.85	3.61
Triglyceride (<1.7 mmol/L)	2.75	–	–	–	–	–	–	–	–	–
Total cholesterol (<5.72 mmol/L)	3.38	–	–	–	–	–	–	–	–	–
Total bilirubin (3.4~17.1 umol/L)	13.5	–	–	–	–	–	–	–	36.0	–
High density lipoprotein (0.9~2.0 mmol/L)	0.61	–	–	–	–	–	–	–	–	–
Low density lipoprotein (0.9~2.0 mmol/L)	1.70	–	–	–	–	–	–	–	–	–
Non high density lipoprotein (1.8~4.1 mmol/L)	2.77	–	–	–	–	–	–	–	–	–
Creatinine (45~104 umol/L)	115.0	–	119.0	–	144.0	144.0	150.0	–	138.0	157.0
Blood ketone body (negative)	Negative	–	–	–	–	–	–	–	–	–
Blood ammonia (9~30 umol/L)	72.48	–	–	–	–	–	–	–	–	–
Erythrocyte sedimentation rate (0.0~15.0 mm/h)	29	–	–	–	–	–	–	–	–	–
Glycosylated hemoglobin Hba1C (4~6%)	7.0	–	–	–	–	–	–	–	–	–
2 h blood glucose (<7.8 mmol/l)	8.32	–	–	–	–	–	–	–	–	–
High sensitive C reactive protein (0~3 mg/L)	2.9	–	–	–	–	–	–	–	–	–
B type natriuretic peptide (0.0~100 pg/mL)	188.00	160.0	–	–	–	–	–	–	–	–
**MYOCARDIAL INFARCTION MARKERS**										
Troponin (<0.04 ng/mL)	0.01	–	–	–	–	–	–	–	10.65	4.01
Creatine kinase (0.6~6.3 ng/mL)	0.7	–	–	–	–	–	–	–	639	–
Myoglobin (17.4~105.7 ng/mL)	33.10	–	–	–	–	–	–	–	–	–
**BLOOD COAGULATION ANALYSIS**										
Fibrin degradation product (0~5 μg/ml)	5.90	10.10	–	–	–	–	–	–	–	–
Thrombin time (14~21 s)	20.50	20.70	–	–	–	–	–	–	–	–
Prothrombin time (9.4~12.5 s)	12.00	14.70	–	13.5	18.50	36.00	37.30	11.4	–	–
Fibrinogen (2.00~4.00 g/L)	3.75	3.67	–	–	–	–	–	–	–	–
Activated partial prothrombin time (25~33.8 s)	28.20	30.10	–	–	–	–	–	–	–	–
Prothrombin INR (0.8~1.15)	1.01	1.23	–	1.13	1.65	2.99	3.10	1.03	–	–
D–Dimer (0~0.5 μg/mL)	0.95	1.48	–	–	–	–	–	–	–	–
**ECHOCARDIOGRAPHY**										
Internal diameter of the aortic root (28~40 mm)	37	–	–	–	–	–	–	–	–	–
Left atrial diameter (30~40 mm)	46	–	–	–	–	–	–	–	–	–
The left ventricular ejection fraction (55%)	52	–	–	–	–	–	–	–	–	–
Left ventricular end diastolic diameter (38~52 mm)	48	–	–	–	–	–	–	–	–	–

*Data were collected from Nov. 8th 2017 to Nov. 30th 2017. Nov., November; -, No tested*.

## Investigations

Clinical investigations were performed to assess the causes of potential decreased dabigatran effects at therapeutic doses. They included patient's characteristic, co-administrated drugs, and genotyping of *ABCB1* and *CES1*.

### *ABCB1* and *CES1* genotyping

Genomic DNA was extracted from whole blood (200 μl) using the QIAamp DNA blood mini kit (QIAGEN, Hombrechtikon, Switzerland). *ABCB1* (rs4148738, rs2235046, rs1128503, rs10276036, rs1202169, rs1202168, and rs1202167) and *CES1* (rs8192935, rs2244613, and rs4122238) and CES1P2 (rs4580160 and rs4784563) polymorphisms were determined by Sanger sequencing. The polymerase chain reaction primers were presented in Table 3. Sequencing was performed on 1 μl of the purified mixture using the BigDye Terminator v1.1 Cycle Sequencing kit (Life Technologies, Warsaw, Poland) and an ABI 3130 Automatic Capillary DNA Sequencer.

**Table 3:** The polymerase chain reaction primers of ABCB1 and CES1.

**SNP**	**Forward**	**Reverse**
rs4148738	TGCTGTTTGTGAGGCCC TTTGCC	TTTTGGTACATTAAAGAATTT GCCATC
rs2235046	AATTAGAAAATGCAGCTG	TCTTGTCAGGTTCTGAGTACC
rs1128503	GAGTTTCTGATGTTTTCTTG	GACCCTGCGGTGATCAGCAG
rs10276036	TTGTGGAGAGCTGGATAAAGTG	AGCCCAGGAGGTAGAGGTTATG
rs1202169	AGTGGTCTCTTTGGAAAAGG	GTAGAAACTTCTACCCTGC
rs1202168	AGTGGTCTCTTTGGAAAAGG	GTAGAAACTTCTACCCTGC
rs1202167	TCTGTCACCCAGGCTGGAGT	TGGTGGGTCTTACCTGATGC
rs8192935	TTATATTATTAAAAACATC	CATTGTTCTCCTCAGGAAT
rs4580160	CCATGCTAAGTATGTAGGGG	CATCCTTCTGAGATTTTCTG
rs4784563	ACTGCCACAGCTTCTCCAC	TCACCTACCTCCCAGCATA
rs2244613	ATCAGCCTTTGAGGCCTGAC	CTGCTAAAAAAAAAAAAAAGT
rs4122238	GAGTGGAGGCGTGGTGGGAG	CCTTCACCCACAACATGCCC

*SNP, single nucleotide polymorphism*.

## Results

Several reasons that contributed to treatment failure with dabigatran should be considered in this case: (1) the patient is a 70-year-old Chinese male and had a moderate renal insufficiency (estimated glomerular filtration rate of 55 mL/min); (2) the drug-drug interaction between dabigatran and atorvastatin was present; (3) as shown in Table 4, The patient is a heterozygote carrier of *ABCB1* variant alleles with 7 heterozygote single nucleotide polymorphisms (SNPs: rs4148738, rs2235046, rs1128503, rs10276036, rs1202169, rs1202168, rs1202167) as well as *CES-1* variant alleles with 2 homozygote SNPs (rs2244613 and rs4122238) and 2 heterozygote SNPs (rs8192935 and rs4580160).

**Table 4:** The results of ABCB1 and CES1 Genotyping.

**SNP**	**Chromosome**	**Position, bp**	**Locus**	**Function**	**Results**	**MAF(allel)**	**MAF in china**
rs4148738	7	87000985	ABCB1	Intron	GA	0.38 (A)	0.41
rs2235046	7	87012002	ABCB1	Intron	AG	0.44 (G)	0.69
rs1128503	7	87017537	ABCB1	Synonymous	TC	0.42 (C)	0.69
rs10276036	7	87018134	ABCB1	Intron	CT	0.43 (T)	0.69
rs1202169	7	87033786	ABCB1	Intron	AG	0.43 (G)	0.69
rs1202168	7	87033898	ABCB1	Intron	CT	0.43 (T)	0.69
rs1202167	7	87034995	ABCB1	Intron	GA	0.43 (A)	0.69
rs8192935	16	54419295	CES1	Intron	AG	0.42 (G)	0.76
rs4580160	16	54326141	CES1P2	Intron	TC	0.50 (C)	0.42
rs4784563	16	54333986	CES1P2	Intron	GG	0.42 (A)	0.24
rs2244613	16	54402110	CES1	Intron	TT	0.33 (T)	0.62
rs4122238	16	54414218	CES1	Intron	GG	0.27 (G)	0.52

*SNP, single nucleotide polymorphism; MAF, minor allele frequency*.

## Discussion

We described the case of an old dabigatran-treated patient who presented with left atrial appendage thrombus formation despite the 31 months uninterrupted dabigatran therapy. Laboratory investigations showed a serum creatinine of 115 μmol/L and estimated glomerular filtration rate of 55 mL/min, which indicated a moderate renal insufficiency. Given that more than 80% of dabigatran is eliminated via urine, renal insufficiency was probably a contributing factor to increase the susceptibility of dabigatran. Furthermore, dabigatran may not suitable for elderly person due to insufficient renal function and correspondingly increased risk of bleeding. However, the mutation of both *ABCB1* and *CES-1* alleles may lead to a significant decrease of dabigatran blood concentration. The *ABCB1* gene encodes for *P-gp*, and *P-gp* is an ATP-dependent drug efflux pump (Verhalen et al., 2017). Dabigatran etexilate, but not dabigatran, is a *P-gp* substrate. *P-gp* inhibitors increase dabigatran bioavailability by 10–20% (Stangier and Clemens, 2009). As compared to the homozygotes for *ABCB1* SNP rs4148738 (G allele), patients carrying 1 or 2 A alleles showed 5% lower trough concentration (Dimatteo et al., 2016). *CES-1* gene, which encodes for the liver carboxylesterase 1 enzyme, is responsible for the biotransformation of dabigatran etexilate into the active metabolite, namely, dabigatran (Wadkins et al., 2001; Redinbo et al., 2003). Paré et al. (2013) conducted a genome-wide association study, and found that the association of *ABCB1* and *CES-1* SNPs was consistent with its effect on dabigatran blood concentration. Of which, *CES-1* SNP rs2244613 is associated at genome-wide significance with trough concentration, while rs8192935 and rs4148738 are associated with peak concentration modestly (Paré et al., 2013; Ross and Paré, 2013). The minor allele of the *CES-1* SNP rs8192935 is associated with a 12% decrease in adjusted peak concentration (Paré et al., 2013). The *CES-1* SNP rs2244613 is associated with a 15% decrease in adjusted trough concentration per minor allele (Paré et al., 2013). Thus, the subject carrying 2 minor alleles of rs2244613 are expected to have 28% lower concentration than no-carriers (Paré et al., 2013). In agreement with genome-wide association study, Gouin-Thibault I et al. (Gouin-Thibault et al., 2016) indicated that the heterozygous and homozygous of rs2244613 (*CES-1*) mutated groups had 14 and 26% lower *AUC* values as well as 13 and 43% lower *C*_max_ values than the wild-type groups. Of note, the frequency of the T allele of rs2244613 (*CES-1*) is 33% in overall populations, but affects up to over 60% in Asians. Also, the frequency of the A allele of rs8192935 affects up to 76% in Asians while that is 42% in overall populations (Table 3). Therefore, the impacts on dabigatran concentrations related to rs2244613 and rs8192935 may be greater than previously postulated especially in Asians. Regretfully, no studies that focused on the relationship between *CES-1* SNP and ischemic events have been carried out. The present case indicated the presence of *ABCB1* heterozygotes in all tested allele. Meanwhile, there is a homozygous mutation of *CES-1* rs2244613 and rs4122238 as well as heterozygote mutation of *CES-1* rs8192935 and rs4580160. Presumably, patient carrying all these alleles may bring about the relatively low dabigatran blood concentration, subsequently leading to thrombus in the left atrial appendage. In addition, the presence of drug interaction with atorvastatin was probably another contributing factor to increase the risk of thrombosis. Atorvastatin is known as a moderate inhibitors of P-gp, which leads to an approximate 20% decrease in dabigatran concentrations (Wessler et al., 2013; Stöllberger and Finsterer, 2015). According to a large retrospective cohort study of 91,330 Taiwanese patients with non-valvular atrial fibrillation who were treated with dabigatran (49.65% of subjects) or another NOACs, concurrent use of atorvastatin was associated with a 29% decrease in the incidence rate ratio of major bleeding (Chang et al., 2017).

At present, there are limited data on the thrombosis in atrial fibrillation with the management of dabigatran. Luis et al. (2013) firstly reported the images which demonstrated the documented case of thrombosis in atrial fibrillation with coexistent valvular heart disease after 4 months anticoagulation with dabigatran. Sharma et al. (2014) report 2 cases of development of large left atrial thrombus in spite of the continuous treatment with dabigatran. Shah et al. (2015) reported another patient with thrombus formation in the left atrium. In all four cases, the formation of thrombus were in the left atrium but not in the left atrial appendage (Shah et al., 2015). Several proposed mechanisms might explain the reason of left atrial thrombus formation on dabigatran therapy. Firstly, dabigatran therapy is a single level downstream inhibition of thrombin, which could lead to a compensatory increase in upstream clotting factors. Secondly, incomplete inhibition of all available coagulation factors leaves some thrombin activity uninhibited or active. Thirdly, potential drug-drug interactions or inadequate absorption may lead to the unachieved therapeutic levels. Currently, the best strategy of anticoagulation in patients who develop thrombus on dabigatran therapy is uncertain, and a tried therapy with warfarin or left atrial appendage closure should be considered in such patients.

Our report had a few potential limitations. Firstly, our study was underpowered to detect the dabigatran plasma concentration. Secondly, we did not investigate *ABCB2* gene polymorphism, which may also impact the blood concentration of dabigatran. Moreover, we did not evaluate *P-gp* and *CES* activity *in vivo*.

## Concluding remarks

We suggest that the presence of *ABCB1* variant alleles and *CES-1* variant alleles and drug-drug interaction with atorvastatin are possibly contributing factors for dabigatran therapy failure in this case. Indeed, dabigatran may not suitable for elderly person owing to insufficient renal function and potential drug-drug interaction. Regarding optimal strategy, the treatment with warfarin or left atrial appendage closure may be the viable regimens in such patients suffering dabigatran therapy failure.

More clinical data are required to elucidate the impact of genetic polymorphism on dabigatran pharmacokinetics and thrombosis formation in atrial atrium or left atrial appendage. The impact of *ABCB1* and *CES-1* gene polymorphism on dabigatran pharmacokinetics should be investigated in a phase 1 clinical study in healthy volunteers based on different races.

## Author contributions

LS was in charge of the treatment of patient. X-YZ was responsible for collecting the patient's information. X-WM was performed the phenotyping test of ABCB1 and CES-1 gene. Z-CG was involved in the care of the patient and interpreted the results, and wrote the manuscript. HL supervised the investigations and interpreted the results, and wrote the manuscript. F-HS was redacted of the manuscript. All authors read and approved the manuscript.

### Conflict of interest statement

The authors declare that the research was conducted in the absence of any commercial or financial relationships that could be construed as a potential conflict of interest.
